# Fumarylacetoacetate Hydrolase Regulates Seed Dormancy and Germination Through the Gibberellin Pathway in Arabidopsis

**DOI:** 10.3390/plants14213342

**Published:** 2025-10-31

**Authors:** Chao Hu, Hua Yang, Xuewen Zhang, Chunmei Ren, Lihua Huang

**Affiliations:** College of Bioscience and Biotechnology, Hunan Agricultural University, Changsha 410128, China; huchao@hunau.edu.cn (C.H.);

**Keywords:** abscisic acid, gibberellin pathway, seed dormancy, seed germination, *SHORT-DAY SENSITIVE CELL DEATH 1*, Tyr degradation pathway

## Abstract

Tyrosine (Tyr) degradation is a crucial pathway in animals. However, its role in plants remains to be examined. Fumarylacetoacetate hydrolase (FAH) is the final enzyme involved in Tyr degradation. Studies of a mutant of the *SHORT-DAY SENSITIVE CELL DEATH 1* (*SSCD1*) gene encoding FAH in Arabidopsis have shown that blockage of this pathway results in the accumulation of Tyr metabolites, thereby inducing cell death under short-day conditions. Seed dormancy is a critical trait which is regulated by endogenous and environmental cues, among which abscisic acid (ABA) and gibberellin (GA) are the primary effectors. ABA induces seed dormancy, whereas GA releases seed dormancy. In this study, *sscd1* seeds displayed deep dormancy and hypersensitivity to the GA biosynthesis inhibitor paclobutrazol, but not to ABA during germination. However, exogenous GA_3_ could not completely recover dormancy or germination of *sscd1* seeds. Moreover, GA_3_ level was reduced, which was consistent with the decreased expression of *GA3-oxidase 1* in imbibed *sscd1* seeds. Furthermore, *SSCD1* acted upstream of *RGA-LIKE 2*. Eliminating the accumulation of Tyr metabolites by inhibiting homogentisate dioxygenase, an enzyme upstream of FAH, completely rescued the phenotype of *sscd1* seeds. Additionally, germination of *sscd1* seeds was hypersensitive to FAH deficiency-induced accumulation of succinylacetone, which is a Tyr metabolite. These findings suggest that FAH deficiency in *sscd1* causes accumulation of Tyr metabolites, thereby disrupting GA biosynthesis and signaling. This resulted in deep dormancy and hypersensitivity to paclobutrazol during germination and highlights the important role of the Tyr degradation pathway in GA-mediated seed dormancy and germination.

## 1. Introduction

Seed dormancy prevents or delays germination even under favorable conditions. The most common class of seed dormancy is physiological dormancy, which is induced during seed maturation and peaks in freshly harvested seeds [1]. Physiological dormancy in Arabidopsis seeds can be released by imbibition at low temperatures (stratification) or dry storage [2,3,4]. Phytohormones, abscisic acid (ABA) and gibberellins (GAs), play essential roles in regulating physiological dormancy and germination. ABA induces seed dormancy and represses germination. Therefore, elevated ABA biosynthesis enhances seed dormancy, whereas decreased ABA biosynthesis releases seed dormancy [1,2]. 9-cis-epoxycarotenoid dioxygenase (NCED) catalyzes carotenoid cleavage, a key step in ABA biosynthesis. In *Arabidopsis thaliana*, *NCED6* and *NCED9* participate in ABA synthesis in seeds. Mutations in *NCED6* and *NCED9* reduce ABA levels in seeds, while overexpression of *NCED6* results in ABA overproduction [5]. Accordingly, shallow and deeper dormancy is observed in *nced6 nced9* mutant seeds or seeds overexpressing *NCED6*, respectively [5]. ABA initiates a signaling pathway by activating SNF1-related protein kinases 2, which phosphorylate downstream transcription factors, such as ABA INSENSITIVE (ABI) 3 and 4 [6]. ABA signaling components regulate seed dormancy. For example, loss of function of *ABI3* or *ABI4* reduced seed dormancy [7,8]. In contrast to ABA, GA promotes the release of seed dormancy and germination [1,2]. GA20-oxidase (GA20ox) and GA3-oxidase (GA3ox) catalyze the final two steps in GA biosynthesis [9]. The major genes in Arabidopsis that produce GA during seed germination are *GA20ox1*, *GA20ox2*, *GA3ox1*, and *GA3ox2* [10,11]. GA2-oxidase (GA2ox) catalyzes the deactivation of bioactive GA [12]. In Arabidopsis, *GA2ox2* and *GA2ox6* regulate seed germination [12,13]. Hence, altered expression of these genes affects germination [10,11,12,13]. GA forms a complex that triggers degradation of DELLA proteins, thereby initiating GA signaling pathway [14]. RGA-LIKE 2 (RGL2) is a key DELLA protein related to seed dormancy and germination. Loss-of-function in *RGL2* reduces seed dormancy and allows germination under GA-limited conditions, whereas *RGL2* overexpression strongly inhibits seed germination [15,16].

The level of Tyr tends to increase during the late stage of seed development in which seed dormancy is established [17]. This suggests that Tyr metabolism may be involved in regulating dormancy. Tyr is degraded to fumarate and acetoacetate in five enzymatic steps. Tyr is first transformed into maleylacetoacetate by Tyr aminotransferase, 4-hydroxyphenylpyruvate dioxygenase, and homogentisate dioxygenase (HGO). Maleylacetoacetate is then metabolized to fumarate and acetoacetate by maleylacetoacetate isomerase and fumarylacetoacetate hydrolase (FAH) [18,19,20]. Tyr degradation is a crucial pathway in animals [18]. Mutations affecting enzymes in this pathway lead to genetic diseases, such as tyrosinemia type I, which is a devastating disorder caused by FAH deficiency [21]. FAH deficiency causes cellular accumulation of Tyr metabolites such as succinylacetone (SUAC), which consequently damages DNA and proteins and results in organ dysfunction [21,22]. Suitably, HGO inactivation protects against the effects of FAH deficiency [21]. Arabidopsis possesses three genes encoding putative FAH domain-containing (FAHD) proteins: *AT3G16700*, *AT1G12050* and *AT4G159409* (*AtFAHD1a*), among which the protein encoded by *AT1G12050* has been confirmed as FAH enzyme [23,24]. However, enzymatic activities of putative proteins encoded by *AT3G16700* and *AtFAHD1a* remain to be investigated [23]. Mutation of the *SHORT-DAY SENSITIVE CELL DEATH 1* (*SSCD1*) gene encoding FAH in Arabidopsis resulted in cell death under short-day conditions. *HGO* knockout restored the cell death of the *sscd1* mutant via interception of toxic Tyr metabolites [25,26]. Zhou et al. [27] reported that the Tyr degradation pathway may regulate cell death through the jasmonate signaling pathway in Arabidopsis. In addition, a lack of FAH decreases the seed-setting rate in rice [28]. These data suggest that the Tyr degradation pathway has a critical role in plants. However, current knowledge of Tyr degradation is limited in plants.

To gain more insight into the Tyr degradation pathway in plants, the role of *SSCD1* in seed dormancy and germination was investigated in this study. *SSCD1* showed an essential role in seed dormancy and germination by affecting the GA pathway. Moreover, loss of function of *SSCD1* enhanced seed dormancy and repressed seed germination by affecting GA biosynthesis and signaling. Furthermore, *HGO* mutation suppressed dormancy and germination in *sscd1* seeds. Overall, the study revealed that the Tyr degradation pathway participates in the GA pathway, which regulates seed dormancy and germination.

## 2. Results

### 2.1. The sscd1 Mutant Showed Increased Seed Dormancy

**Figure 1:**
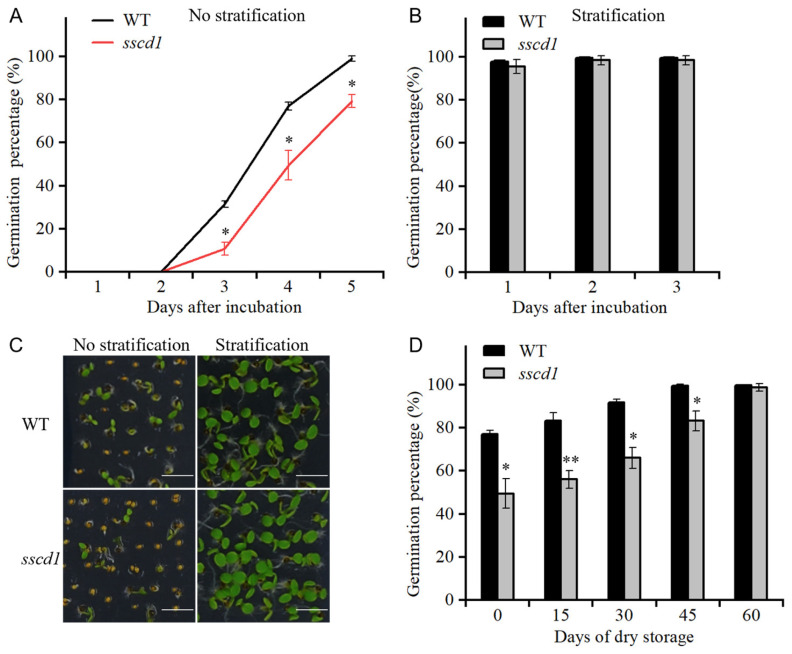
The *sscd1* mutant exhibited increased seed dormancy. (**A**,**B**) Germination percentages of freshly harvested wild type (WT) and *sscd1* seeds without (**A**) or with (**B**) stratification treatment. (**C**) Germination phenotypes of the seeds described in (**A**,**B**) on the 4th day after incubation at 23 °C. Scale bars = 2.78 mm. (**D**) Germination percentages scored four days after incubation, of WT and *sscd1* seeds stored for the time indicated without stratification treatment. Data in (**A**,**B**,**D**) represent the mean ± standard error (SE) from three biological replicates. Seeds were pooled from at least 10 plants, and at least 50 seeds per genotype were used in each replicate. Asterisks represent significant differences between the WT and *sscd1* seeds (*t*-test, * *p* < 0.05, ** *p* < 0.01).

To investigate the effect of *SSCD1* mutation on seed dormancy, seed germination of the *sscd1* mutant and wild-type Col-0 (WT) was monitored. Using freshly harvested seeds, the *sscd1* mutant exhibited delayed seed germination compared with that of the WT without stratification treatment (Figure 1A,C). On the 4th day after sowing, the germination percentage of *sscd1* seeds was <49.5%, whereas that of WT seeds was >77% (Figure 1A). These results indicate that *sscd1* seeds had increased dormancy. Furthermore, the germination of *sscd1* and WT seeds after stratification or dry storage was examined. The germination percentage of freshly harvested *sscd1* seeds was comparable with that of freshly harvested WT seeds after three days of cold stratification in darkness at 4 °C (Figure 1B,C). When stored for 30 days at 23 °C, only 66% of *sscd1* seeds germinated, whereas 91.7% of the WT seeds germinated on the 4th day after sowing (Figure 1D). When stored for 60 days, the mutant seeds exhibited no significant differences from the WT seeds during germination (Figure 1D). These results demonstrated that stratification treatment and dry storage can break *sscd1* seed dormancy.

### 2.2. The sscd1 and WT Seed Showed Similar Responses to ABA

The germination of *sscd1* seeds stored for 15 days under ABA treatment was investigated. The *sscd1* mutant did not exhibit a significant difference in sensitivity to ABA compared with that of the WT during germination. Moreover, the ABA content in *sscd1* and WT seeds either before or 24 h after seed imbibition was not significantly different. In addition, no obvious differences were observed in terms of the expression of ABA biosynthesis and signaling genes (*NCED6*, *NCED9*, *ABI3*, and *ABI4*) in both dry and imbibed seeds of *sscd1* and WT plants (Figure S1). These data suggested that *SSCD1*-regulated dormancy does not involve the ABA signaling pathway.

### 2.3. Dormant sscd1 Seeds Were Less Sensitive to GA

**Figure 2:**
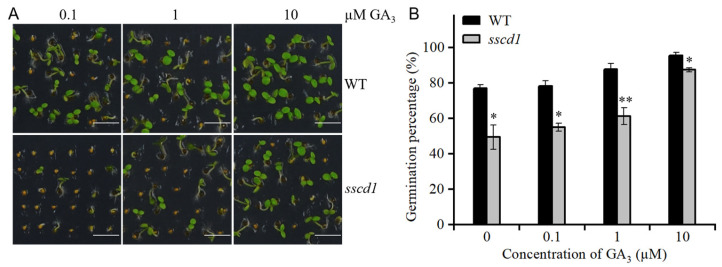
The *sscd1* mutant showed reduced sensitivity to GA. (**A**) Germination phenotypes of freshly harvested WT and *sscd1* seeds on the 4th day after incubation in the presence of different GA_3_ concentrations without stratification treatment. Scale bars = 2.78 mm. (**B**) Germination percentages of WT and *sscd1* seeds described in (**A**). Data represent the mean ± SE from three biological replicates. Seeds were pooled from at least 10 plants, and at least 50 seeds per genotype were used in each replicate. Asterisks represent significant differences between the WT and *sscd1* seeds (*t*-test, * *p* < 0.05, ** *p* < 0.01).

To examine whether GA is involved in seed dormancy in the *sscd1* mutant, the germination of freshly harvested seeds was evaluated in the presence of GA_3_. As shown in Figure 2, the germination percentage of *sscd1* seeds was obviously lower than that of WT seeds after GA_3_ treatment. In the presence of 1 µM GA_3_, *sscd1* seeds showed 61.3% germination, whereas WT seeds showed 87.8% germination on the 4th day after sowing. In the presence of 10 µM GA_3_, *sscd1* germinated on average 8.7% less than WT (Figure 2). These results confirm that dormant *sscd1* seeds are less responsive to GA, thus suggesting that GA signaling is involved in *SSCD1*-regulated seed dormancy.

### 2.4. SSCD1 Mutation Affects GA Biosynthesis

**Figure 3:**
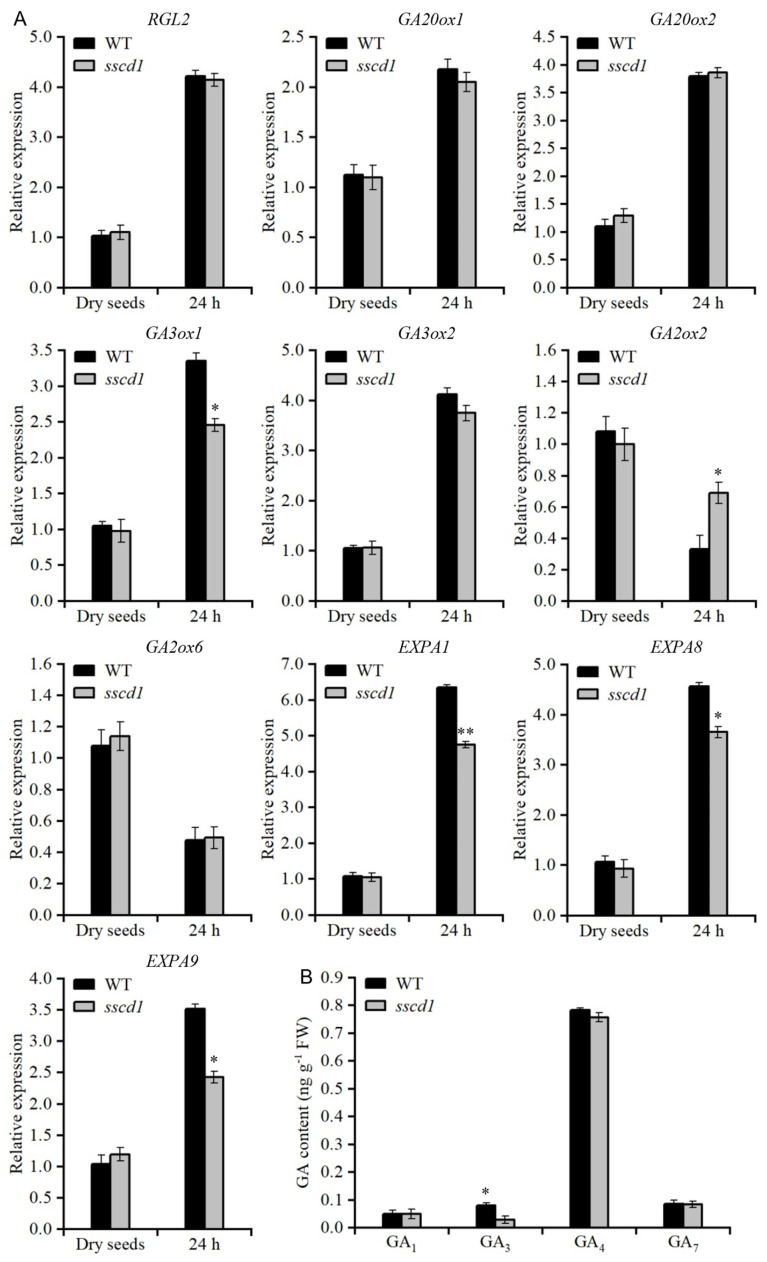
The *sscd1* mutant showed decreased GA biosynthesis. (**A**) Relative expression of genes involved in GA signaling (*RGL2*), biosynthesis (*GA20ox1*, *GA20ox2*, *GA3ox1*, *GA3ox2*), catabolism (*GA2ox2*, *GA2ox6*), and cell wall extension (*EXPA1*, *EXPA8*, *EXPA9*) in freshly harvested dry seeds and seeds imbibed for 24 h on MS medium from WT and *sscd1* plants. *RGL2*, *RGA-LIKE 2*. *GA20ox1*, *GA20-oxidase 1*. *GA20ox2*, *GA20-oxidase 2*. *GA3ox1*, *GA3-oxidase 1*. *GA3ox2*, *GA3-oxidase 2*. *GA2ox2*, *GA2-oxidase 2*. *GA2ox6*, *GA2-oxidase 6*. *EXPA1*, *expansin A1*. *EXPA8*, *expansin A8*. *EXPA9*, *expansin A9*. (**B**) GA_1_, GA_3_, GA_4_, and GA_7_ content in freshly harvested WT and *sscd1* seeds imbibed for 24 h on MS medium. All data represent the mean ± SE from three biological replicates. Asterisks represent significant differences between the WT and *sscd1* seeds (*t*-test, * *p* < 0.05, ** *p* < 0.01).

As previously mentioned, *sscd1* seeds displayed a reduced response to GA (Figure 2). RGL2 is a critical repressor of the GA response [14]. Therefore, *RGL2* expression in the dry and imbibed seeds of *sscd1* and WT plants was compared. *RGL2* showed no differences in *sscd1* seeds compared with that in WT seeds (Figure 3A). GA biosynthesis is subject to negative feedback regulation via the GA response [29,30]. Therefore, expression of *GA20ox1*, *GA20ox2*, *GA3ox1* and *GA3ox2* in dry and imbibed seeds from *sscd1* and WT plants was analyzed. No significant difference in *GA20ox1*, *GA20ox2*, and *GA3ox2* expression was found between *sscd1* and WT seeds. However, decreased *GA3ox1* expression was detected in imbibed *sscd1* seeds (Figure 3A). Moreover, increased expression of *GA2ox2* was detected in imbibed *sscd1* seeds (Figure 3A). Endogenous GA content was also measured in freshly harvested seeds of *sscd1* and WT plants imbibed for 24 h. GA_3_ level was lower in *sscd1* seeds than in WT seeds (Figure 3B). Furthermore, some GA-regulated genes involved in cell wall extension during germination, such as expansin (*EXP*) *A1*, *EXPA8*, and *EXPA9,* were expressed at markedly lower levels in imbibed *sscd1* seeds than in imbibed WT seeds, although their expression levels were similar in dry seeds (Figure 3A). These data suggest that *SSCD1* mutation repressed GA biosynthesis in imbibed seeds.

### 2.5. The sscd1 Seeds Were Hypersensitive to Paclobutrazol

**Figure 4:**
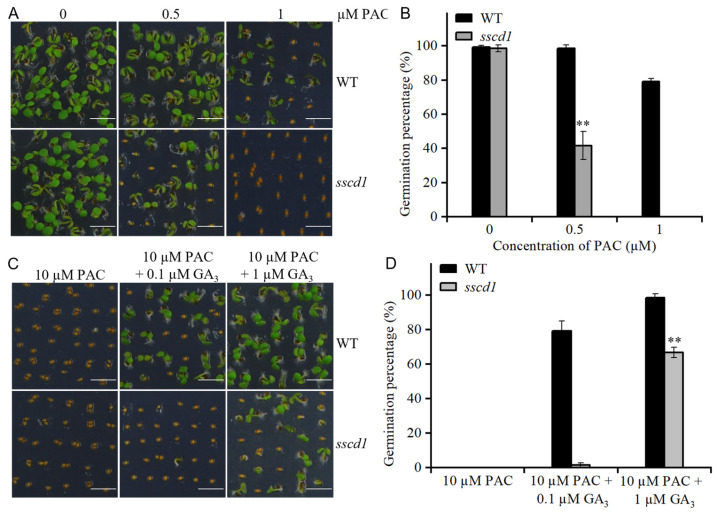
The *sscd1* mutant showed increased sensitivity to paclobutrazol (PAC). (**A**) Germination phenotypes of WT and *sscd1* seeds after 60 days of storage on the 4th day after stratification in the presence of different PAC concentrations. Scale bars = 2.78 mm. (**B**) Germination percentages of WT and *sscd1* seeds described in (**A**). (**C**) Germination phenotypes of WT and *sscd1* seeds on the 4th day after stratification in the presence of 10 µM PAC and 0.1 or 1 µM GA_3_. Scale bars = 2.78 mm. (**D**) Germination percentages of WT and *sscd1* seeds described in (**C**). Data in (**B**,**D**) represent the mean ± SE from three biological replicates. Seeds were pooled from at least 10 plants, and at least 50 seeds per genotype were used in each replicate. Asterisks represent significant differences between the WT and *sscd1* seeds (*t*-test, ** *p* < 0.01).

To further verify the role of *SSCD1* in GA-mediated seed germination, the germination of *sscd1* and WT seeds after 60 days of storage was investigated in the presence of paclobutrazol (PAC). The *sscd1* seeds exhibited PAC hypersensitivity. In the presence of 0.5 µM PAC on the 4th day after sowing, *sscd1* seeds germinated 41.7%, whereas WT seeds germinated 98.5% (Figure 4A,B). Germination of *sscd1* seeds was completely inhibited in the presence of 1 µM PAC (Figure 4A,B). Germination of *sscd1* and WT seeds was also evaluated in the presence of 10 µM PAC and various GA_3_ concentrations. As shown in Figure 4C,D, in the presence of 0.1 µM GA_3_, PAC-treated *sscd1* seeds showed 1.6% germination, whereas PAC-treated WT seeds showed 79.2% germination on the 4th day after sowing. In the presence of 1 µM GA_3_, *sscd1* germinated on average 32.3% less than WT (Figure 4C,D). Moreover, 10 µM GA_3_ treatment improved germination of PAC-treated *sscd1* and WT seeds. Although the germination percentage of *sscd1* seeds was comparable with that of WT seeds on the 2nd day after sowing, the germination percentage of *sscd1* seeds was obviously lower than that of WT seeds on the first day after sowing (Figure S2). These results showed that defects in GA biosynthesis and signaling were responsible for the strong *sscd1* germination inhibition in the presence of PAC.

### 2.6. GA Biosynthesis and Cell Wall Extension Genes Were Modulated in sscd1 Seeds Under PAC Treatment

**Figure 5:**
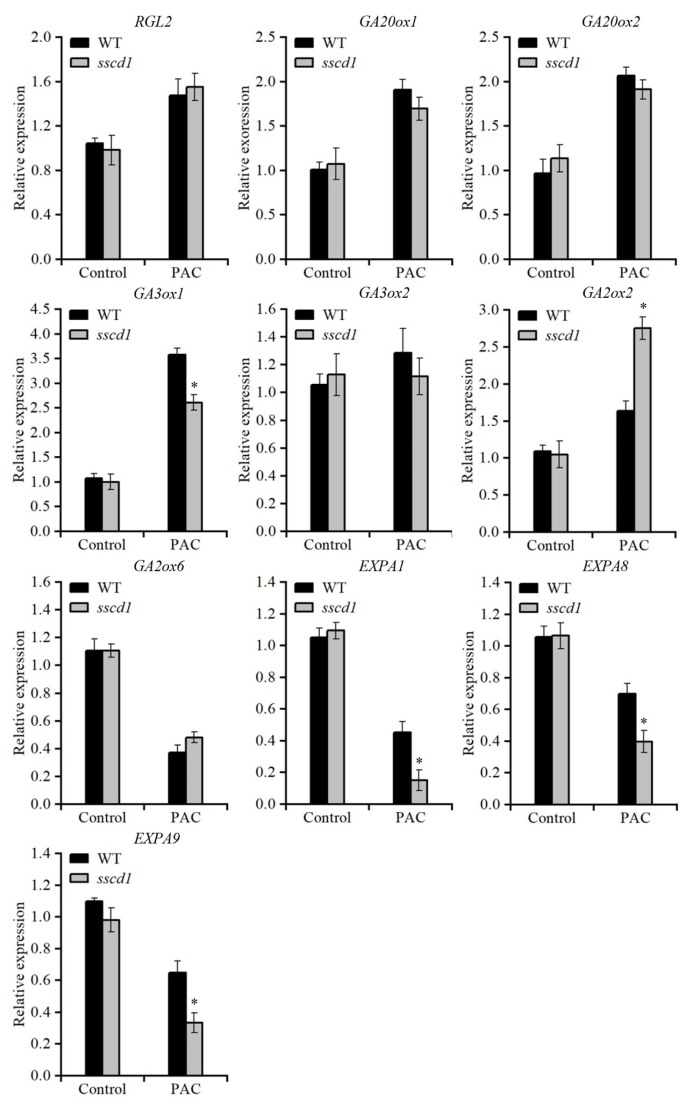
Expression analysis of genes involved in the GA pathway in WT and *sscd1* seeds treated with paclobutrazol (PAC). Relative expression of genes involved in GA signaling (*RGL2*), biosynthesis (*GA20ox1*, *GA20ox2*, *GA3ox1*, *GA3ox2*), catabolism (*GA2ox2*, *GA2ox6*), and cell wall extension (*EXPA1*, *EXPA8*, *EXPA9*) in WT and *sscd1* seeds imbibed for 12 h on MS medium without PAC (control) or with 10 µM PAC after stratification. *RGL2*, *RGA-LIKE 2*. *GA20ox1*, *GA20-oxidase 1*. *GA20ox2*, *GA20-oxidase 2*. *GA3ox1*, *GA3-oxidase 1*. *GA3ox2*, *GA3-oxidase 2*. *GA2ox2*, *GA2-oxidase 2*. *GA2ox6*, *GA2-oxidase 6*. *EXPA1*, *expansin A1*. *EXPA8*, *expansin A8*. *EXPA9*, *expansin A9*. Data represent the mean ± SE from three biological replicates. Asterisks represent significant differences between the WT and *sscd1* seeds (*t*-test, * *p* < 0.05).

To gain further insights into the relationship between *SSCD1*-regulated seed germination and the GA pathway, the expression of GA pathway-related genes in PAC-treated *sscd1* and WT seeds was analyzed. In the absence of PAC, these genes showed similar expression in imbibed *sscd1* and WT seeds (Figure 5). In the presence of PAC, the expression of *RGL2* was similar in *sscd1* and WT seeds. Among the GA synthesis genes, *GA3ox1* was downregulated, whereas *GA2ox2* was upregulated in *sscd1* seeds under PAC treatment. *EXPA1*, *EXPA8*, and *EXPA9* were expressed at considerably lower levels in PAC-treated *sscd1* seeds than in PAC-treated WT seeds (Figure 5). The data showed that *SSCD1* mutation alters the expression of genes involved in GA biosynthesis and cell wall extension under PAC-induced inhibition of GA biosynthesis.

### 2.7. SSCD1 Acted Upstream of RGL2

**Figure 6:**
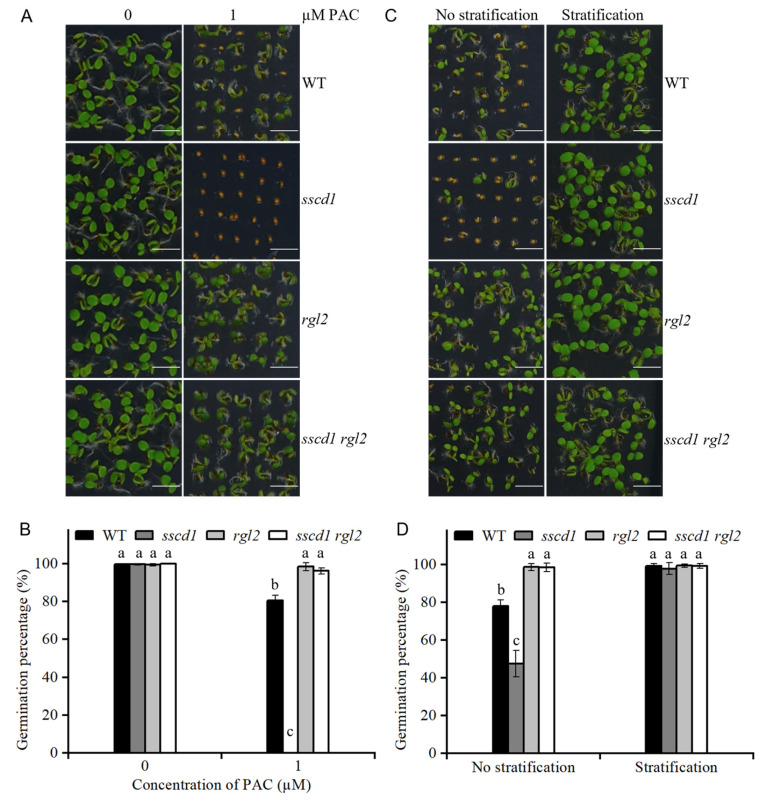
*SSCD1* functions upstream of *RGl2*. (**A**) Germination phenotypes of WT, *sscd1*, *rgl2*, and *sscd1 rgl2* seeds after 60 days of storage on the 4th day after stratification in the presence of 0 and 1 µM paclobutrazol (PAC). Scale bars = 2.78 mm. (**B**) Germination percentages of WT, *sscd1*, *rgl2*, and *sscd1 rgl2* seeds described in (**A**). Data represent the mean ± SE from three biological replicates. Different letters indicate significant differences between the genotypes (Kruskal–Wallis test with Dunn’s post hoc test, *p* < 0.05). (**C**) Germination phenotypes of freshly harvested WT, *sscd1*, *rgl2*, and *sscd1 rgl2* seeds on the 4th day after incubation without stratification treatment. Scale bars = 2.78 mm. (**D**) Germination percentages of WT, *sscd1*, *rgl2*, and *sscd1 rgl2* seeds described in (**C**). Data represent the mean ± SE from three biological replicates. Different letters indicate significant differences between the genotypes (one-way analysis of variance [ANOVA] with Tukey’s post hoc test, *p* < 0.05). Seeds were pooled from at least 10 plants, and at least 50 seeds per genotype were used in each replicate.

These results suggest that GA signaling is involved in seed germination mediated by *SSCD1* (Figure 2 and Figure 4). RGL2 is a negative regulator of GA signaling [14,15,16]. To analyze the genetic relationship between *SSCD1* and *RGL2*, a *sscd1 rgl2* double mutant was generated by crossing the *sscd1* mutant with a *rgl2* mutant [31]. The *rgl2* seeds displayed PAC insensitivity and reduced dormancy during germination (Figure 6). The seed germination phenotype of the double mutant was similar to that of the *rgl2* mutant (Figure 6). These data suggest that *SSCD1* acts upstream of *RGL2*.

### 2.8. Tyr Metabolites Were Involved in SSCD1-Regulated Seed Dormancy and Germination

**Figure 7:**
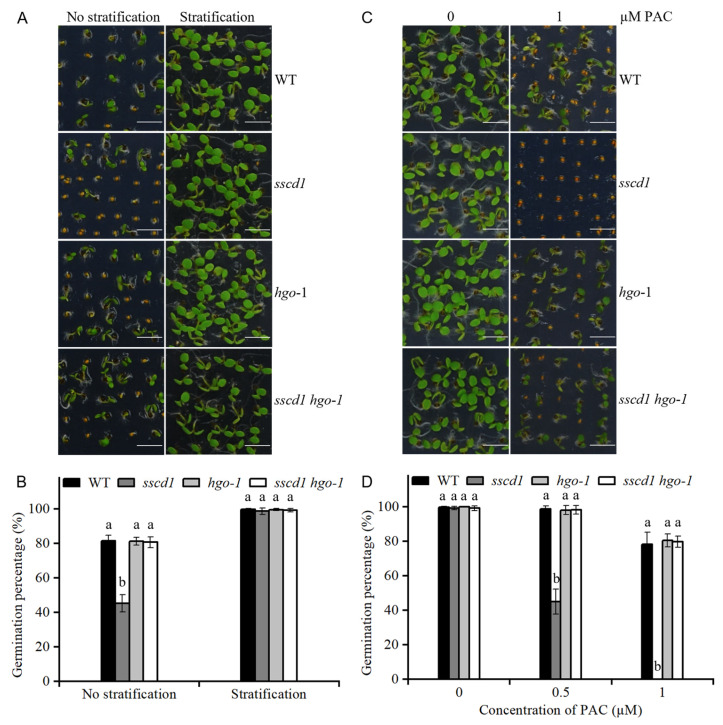
*HGO* mutation completely suppressed deep dormancy and paclobutrazol (PAC) hypersensitivity in *sscd1* seeds. (**A**) Germination phenotypes of freshly harvested WT, *sscd1*, *hgo-1*, and *sscd1 hgo-1* seeds on the 4th day after incubation without stratification treatment. Scale bars = 2.78 mm. (**B**) Germination percentages of WT, WT, *sscd1*, *hgo-1*, and *sscd1 hgo-1* seeds described in (**A**). Data represent the mean ± SE from three biological replicates. Different letters indicate significant differences between the genotypes (one-way ANOVA with Tukey’s post hoc test, *p* < 0.05). (**C**) Germination phenotypes of WT, *sscd1*, *hgo-1*, and *sscd1 hgo-1* seeds after 60 days of storage on the 4th day after stratification in the presence of 0 and 1 µM PAC. Scale bars = 2.78 mm. (**D**) Germination percentages of the WT, *sscd1*, *hgo-1*, and *sscd1 hgo-1* seeds treated with different PAC concentrations. Data represent the mean ± SE from three biological replicates. Different letters indicate significant differences between the genotypes (Kruskal–Wallis with Dunn’s post hoc test, *p* < 0.05). Seeds were pooled from at least 10 plants, and at least 50 seeds per genotype were used in each replicate.

**Figure 8:**
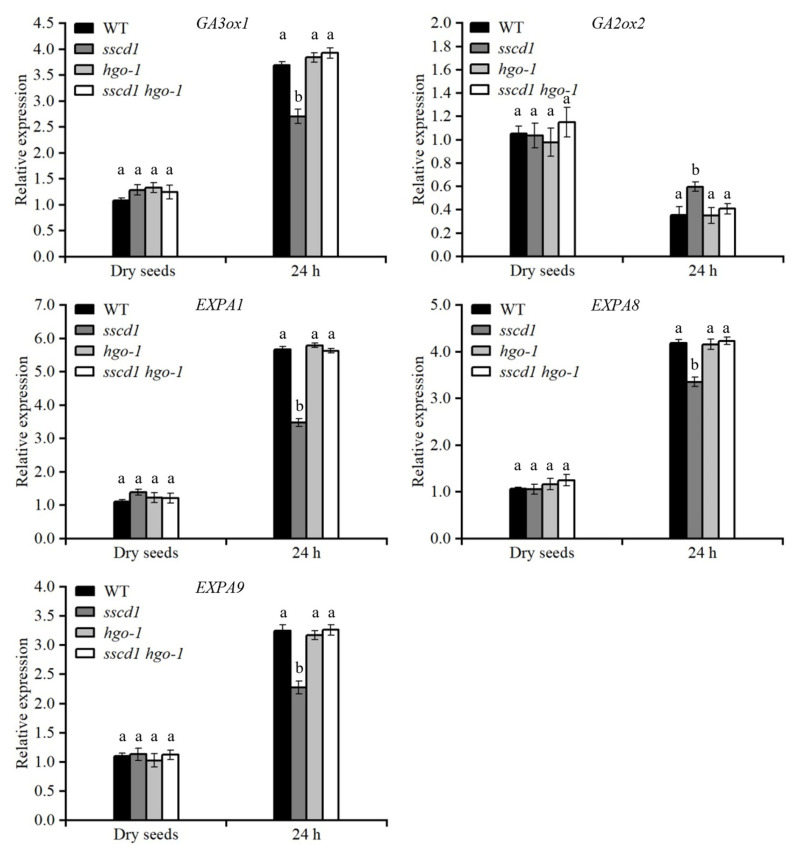
*HGO* mutation suppressed the effect of *sscd1* mutation on gene expression in the GA pathway. Relative expression of genes involved in GA biosynthesis (*GA3ox1*), catabolism (*GA2ox*2) and cell wall extension (*EXPA1*, *EXPA8*, *EXPA9*) in freshly harvested dry seeds and seeds imbibed for 24 h on MS medium from WT, *sscd1*, *hgo-1*, and *sscd1 hgo-1* plants. *GA3ox1*, *GA3-oxidase 1*. *GA2ox2*, *GA2-oxidase 2*. *EXPA1*, *expansin A1*. *EXPA8*, *expansin A8*. *EXPA9*, *expansin A9*. Data represent the mean ± SE from three biological replicates. Different letters indicate significant differences between the genotypes (one-way ANOVA with Tukey’s post hoc test, *p* < 0.05).

**Figure 9:**
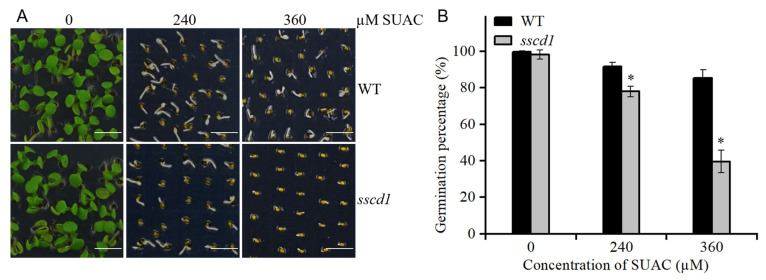
The *sscd1* mutant was hypersensitive to succinylacetone (SUAC) during germination. (**A**) Germination phenotypes of WT and *sscd1* seeds after 60 days of storage on the 6th day after stratification in the presence of different SUAC concentrations. Scale bars = 2.78 mm. (**B**) Germination percentages of WT and *sscd1* seeds described in (**A**). Data represent the mean ± SE from three biological replicates. Seeds were pooled from at least 10 plants, and at least 50 seeds per genotype were used in each replicate. Asterisks represent significant differences between the WT and *sscd1* (*t*-test, * *p* < 0.05).

FAH deficiency causes cellular accumulation of Tyr metabolites, such as fumarylacetoacetate and SUAC, which ultimately stunts growth [21,22]. To test the relationship between these metabolites and *SSCD1*-regulated seed dormancy and germination, the seed germination of the *sscd1 hgo-1* mutant, in which fumarylacetoacetate and SUAC could not be produced, was first analyzed [22,25]. The germination percentage of freshly harvested *sscd1 hgo-1* seeds was comparable with that of freshly harvested WT seeds, with or without stratification (Figure 7A,B). In the presence of PAC, germination of *sscd1 hgo-1* seeds was similar to that of the WT seeds (Figure 7C,D). These results showed that *HGO* mutation suppressed the effect of *SSCD1* mutation on seed dormancy and germination. Additionally, *HGO* mutation rescued the expression of genes associated with GA biosynthesis and cell wall extension in the imbibed *sscd1* seeds (Figure 8). Thereafter, the *sscd1* and WT seeds after 60 days of storage were sown on MS media containing SUAC, and seed germination was assessed. SUAC treatment delayed seed germination, and *sscd1* seeds were more sensitive than WT seeds to SUAC (Figure 9). These results suggest that the accumulation of Tyr metabolites may be responsible for the dormancy and germination phenotypes observed in *sscd1* seeds.

### 2.9. SSCD1 Expression Was Upregulated During Imbibition of Seeds

**Figure 10:**
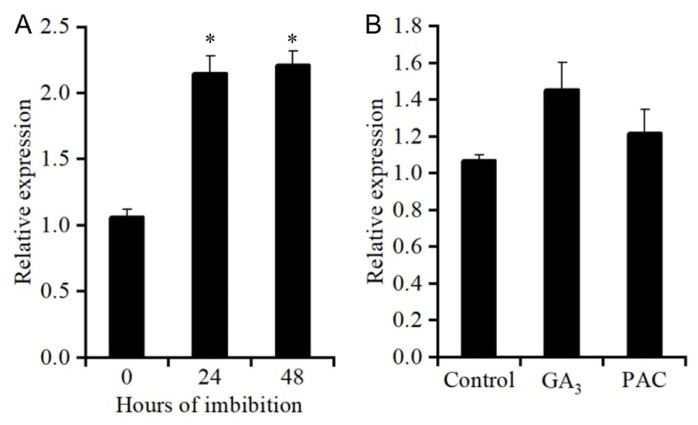
Analysis of *SSCD1* expression. (**A**) Relative expression of *SSCD1* during imbibition. Freshly harvested dry seeds were placed on MS medium for 24 and 48 h at 23 °C. Asterisks represent significant differences compared to dry seeds (0 h) (*t*-test, * *p* < 0.05). (**B**) Response of *SSCD1* to exogenous GA_3_ or PAC in imbibed seeds. Freshly harvested dry seeds were imbibed on MS medium without (control) or with 10 µM PAC or GA_3_ for 24 h at 23 °C. All data represent the mean ± SE from three biological replicates.

To better understand the function of *SSCD1* in seed dormancy and germination, *SSCD1* expression in dry and imbibed seeds was analyzed. *SSCD1* expression was higher in the imbibed seeds than in the dry seeds (Figure 10A). Furthermore, the effects of GA and PAC on *SSCD1* expression were analyzed. As shown in Figure 10B, *SSCD1* expression was not significantly affected by GA_3_ or PAC treatment.

## 3. Discussion

The balance between ABA and GA is important for induction, maintenance and release of dormancy [1]. High endogenous ABA and low GA levels cause deep seed dormancy [1,2]. The Tyr degradation pathway is essential for survival of Arabidopsis under short-day conditions [25]. However, the involvement of this pathway in seed dormancy mediated by the ABA or GA pathway remains unclear. In this study, the loss of function of *SSCD1* enhanced seed dormancy (Figure 1). The response to exogenous ABA during germination, ABA levels in seeds, and expression of key ABA pathway genes were not affected by *SSCD1* mutation (Figure S1). Thus, the seed dormancy regulated by *SSCD1* is not dependent on the ABA pathway. Furthermore, the deep dormant phenotype of freshly harvested *sscd1* seeds was not fully rescued by exogenous GA_3_ application (Figure 2), suggesting that the mutant has defective GA signaling. GA biosynthesis is regulated by GA signaling feedback [29,30]. Some genes, such as *GA20ox1*, *GA20ox2*, and *GA3ox1,* are negatively regulated by the GA response, whereas *GA2ox2* is positively regulated [29,30]. Unexpectedly, *GA3ox1* showed decreased expression in the imbibed *sscd1* seeds, whereas *GA2ox2* had increased expression (Figure 3A). Consistent with the expression of these genes, GA_3_ level was lower in *sscd1* seeds than in WT seeds during imbibition (Figure 3B). These data reveal that *SSCD1* mutation influences GA feedback regulation. During seed imbibition, GA induces the expression of *EXPA* genes, which enables the cell expansion needed for germination [32,33,34,35]. In Arabidopsis, *EXPA1*, *EXPA8* and *EXPA9* are induced by GA and promote GA-mediated germination [32,33,34,35]. These three genes showed decreased expression in imbibed *sscd1* seeds in accordance with the decreased GA_3_ level and dormant phenotypes (Figure 1 and Figure 3). These data suggest that *SSCD1* mutation results in defects in GA biosynthesis and signaling, thus enhancing dormancy in *sscd1* seeds. In addition, dry-stored *sscd1* seeds were hypersensitive to PAC during germination. Exogenous GA_3_ application did not completely recover the restricted germination of *sscd1* seeds under PAC treatment (Figure 4). In the presence of PAC, *EXPA1*, *EXPA8* and *EXPA9* expression was markedly lower in *sscd1* seeds than in WT seeds (Figure 5). Genetic analysis of seed germination in the *sscd1 rgl2* mutant showed that *SSCD1* acts upstream of *RGL2* (Figure 6). These results provide further evidence that repression of GA biosynthesis and signaling results in the germination phenotype observed in *sscd1* seeds.

*HGO* inactivation can prevent the accumulation of Tyr metabolites and cell death caused by FAH mutations [22,25,36]. *HGO* mutation suppressed deep dormancy and PAC hypersensitivity in *sscd1* seeds (Figure 7). The effect of *SSCD1* mutation on the expression of genes in the GA pathway in imbibed seeds was also suppressed by the *HGO* mutation (Figure 8). Furthermore, *sscd1* seeds exhibited increased sensitivity to SUAC inhibition during germination (Figure 9). These data show that deep dormancy and PAC hypersensitivity of *sscd1* seeds are associated with Tyr metabolites. Studies using biological materials from patients and animals with type I tyrosinemia and cultured cells treated with Tyr metabolites have shown that Tyr metabolites affect gene expression and protein functionality [37,38]. For example, SUAC reacts with amino acids and proteins to affect enzyme activity in animals [39]. Moreover, in *sscd1* mutant, these metabolites alter gene expression and enzyme activity [26]. In this study, some genes of the GA pathway analyzed, such as *RGL2*, *GA20ox1* and *GA2ox2*, were not altered in *sscd1* seeds (Figure 3A). The study hypothesized that Tyr metabolites in *sscd1* seeds may modify the expression of genes targeted at the post-transcriptional level or their protein functionality, resulting in reduced GA biosynthesis and signaling.

In summary, FAH deficiency in *sscd1* seeds leads to the accumulation of Tyr metabolites, which inhibit GA biosynthesis and signaling, resulting in increased dormancy and sensitivity of *sscd1* seeds to PAC during germination. This study demonstrates that the Tyr degradation pathway also participates in the GA pathway to regulate seed dormancy and germination. Ultimately, the study further enriches existing knowledge of the role of this pathway in plants.

Studies have shown that FAHD proteins exhibit distinct enzymatic activities and catalyze different reactions in animals [22,40]. Hence, these proteins seem to regulate growth and development in animals by different mechanisms. Gerna et al. [23] reported that *AtFAHD1a* regulates seed dormancy imposed by temperature through affecting seed metabolism such as lysine metabolism. The data suggest that FAHD proteins in Arabidopsis may participate in different pathways to regulate seed dormancy and germination in response to different environmental cues.

## 4. Materials and Methods

### 4.1. Plant Materials and Growth Conditions

*Arabidopsis thaliana* Columbia-0 (Col-0) was used in all the experiments. The *sscd1* mutant with a single nucleotide non-sense mutation, the *hgo-1* and *sscd1 hgo-1* mutants have been previously described by Han et al. [25]. The *rgl2* mutant (SALK_124231) was obtained from the Arabidopsis Biological Resource Center [41]. The *sscd1 rgl2* double mutant was generated by crossing the *sscd1* mutant with the *rgl2* mutant. Plants homozygous for T-DNA insertion at the *RGL2* locus were identified via genotyping using primers (Table S1). The genotype of the *SSCD1* gene in the homozygous double mutant was identified via sequencing. All plants were grown at 23 °C and 70% relative humidity under long-day conditions (16 h light/8 h dark). Seeds were collected from plants grown for 5 weeks in soil, and pooled, then dried for 2 days at 23 °C in darkness. Dry seeds (freshly harvested seeds) were used for germination assays or stored at 23 °C in darkness in closed tubes for up to 60 days before all analyses were completed.

### 4.2. Seed Dormancy and Germination Assays

For seed dormancy assays, seeds were sterilized with 10% sodium hypochlorite solution for 15 min, rinsed with sterile water four times, and then germinated on Murashige and Skoog medium containing 0.7% (*w*/*v*) agar (MS). Seeds were either stratified or not for three days in darkness at 4 °C. For GA sensitivity analysis, freshly harvested seeds were germinated on MS medium containing 0.1, 1.0, or 10 µM GA_3_ without stratification. To assess seed germination under ABA treatment, sterilized seeds stored for 15 days were placed on MS medium containing 0.25, 0.5, or 0.75 µM ABA, and then stratified for three days. To assess seed germination under PAC treatment, seeds after storage for 60 days were sown on MS medium supplemented with 0.5 or 1.0 µM PAC and then stratified for three days. For SUAC responsiveness tests, seeds after storage for 60 days were sown on MS medium containing 240 or 360 µM SUAC and then stratified for three days. All seeds were grown at 23 °C and 70% relative humidity under long-day conditions. Germinated seeds with protruded radicles were scored at the indicated time points. At least 150 seeds from each genotype were used for the three biological replicates.

### 4.3. Gene Expression Analysis

Total RNA was extracted from the seeds using the RNAprep Pure Plant Kit (Tiangen, Beijing, China), following the manufacturer’s protocols. cDNA synthesis and quantitative real-time PCR (qRT-PCR) were performed according to the method of Huang et al. [36]. Product specificity was confirmed using melting curves. *ACTIN2* was used for normalization. The relative expression was calculated by the 2^−ΔΔCt^ method. Three technical and biological replicates were used. The primers used are shown in Table S1.

### 4.4. ABA and GA Measurements

20 mg seeds after 15 days of storage were used for ABA measurements. For GA measurements, freshly harvested seeds were imbibed on MS medium at 23 °C for 24 h in a growth chamber (16 h light/8 h dark). 400 mg imbibed seeds used for GA measurements. Quantification of ABA and GA was performed using gas chromatography-triple-quadrupole tandem mass spectrometry, as described previously [42]. Three biological replicates were analyzed.

### 4.5. Statistical Analysis

Data in all the figures are expressed as the means ± SE. For data that follow a normal distribution and similar variance between groups, statistical differences between mean values were determined using a two-tailed Student’s *t*-test or one-way ANOVA with Tukey’s post hoc test. For data that were not normally distributed or where the variance between groups was not similar, statistical differences between mean values were determined using the Kruskal–Wallis test with Dunn’s post hoc test. Differences at the level of *p* < 0.05 were considered significant.

### 4.6. Accession Numbers

Sequence data from this article can be found in the Arabidopsis Genome Initiative or GenBank under the following accession numbers: AT1G12050 (*SSCD1*), AT5G54080 (*HGO*), AT3G18780 (*ACTIN2*), AT3G24220 (*NCED6*), AT1G78390 (*NCED9*), AT3G24650 (*ABI3*), AT2G40220 (*ABI4*), AT3G03450 (*RGL2*), AT4G25420 (*GA20ox1*), AT5G51810 (*GA20ox2*), AT1G15550 (*GA3ox1*), AT1G80340 (*GA3ox2*), AT1G02400 (*GA2ox6*), AT1G30040 (*GA2ox2*), AT1G69530 (*EXPA1*), AT2G40610 (*EXPA8*), AT5G02260 (*EXPA9*).

## Data Availability

All data in this study are available within the paper and within its Supplementary Materials published online.

## Supplementary Materials

The following supporting information can be downloaded at: https://www.mdpi.com/article/10.3390/plants14213342/s1, Figure S1: Seed germination of WT and *sscd1* in response to ABA. (A) Germination phenotypes of WT and *sscd1* seeds stored for 15 days on the 4th day after stratification in the presence of different ABA concentrations. Scale bars = 2.78 mm. (B) Germination percentages of WT and *sscd1* seeds described in (A). Seeds were pooled from at least 10 plants, and at least 50 seeds per genotype were used in each replicate. (C) ABA contents in dry seeds and seeds imbibed for 24 h on MS medium from WT and *sscd1* plants. (D) Relative expression of genes in the ABA pathway in WT and *sscd1* seeds is described in (C). *NCED6*, *9-cis-epoxycarotenoid dioxygenase 6. NCED9*, *9-cis-epoxycarotenoid dioxygenase 9*. *ABI3*, *ABA INSENSITIVE 3*. *ABI4*, *ABA INSENSITIVE 4*. Data represent the mean ± SE from three biological replicates; Figure S2: Germination of WT and *sscd1* seeds after 60 days of storage in the presence of 10 µM PAC and 10 µM GA_3_. (A) Germination phenotypes of WT and *sscd1* seeds on the 2nd and 4th day after stratification. Scale bars = 2.78 mm. (B) Germination percentages of WT and *sscd1* seeds. Data represent the mean ± SE from three biological replicates. Asterisks represent significant differences between WT and *sscd1* (*t*-test, ** *p* < 0.01). Seeds were pooled from at least 10 plants, and at least 50 seeds per genotype were used in each replicate; Table S1: List of primers used in this study.
