# Supplementary material for: Fumarylacetoacetate Hydrolase Regulates Seed Dormancy and Germination Through the Gibberellin Pathway in Arabidopsis
Source: Plants (Basel). 2025 Oct 31;14(21):3342. doi: 10.3390/plants14213342 (PMC12608377; doi:10.3390/plants14213342)
Supplement: Supplementary file 1 [file plants-14-03342-s001.zip › plants-3884705-Supplementary Figures S1-S2.pdf]

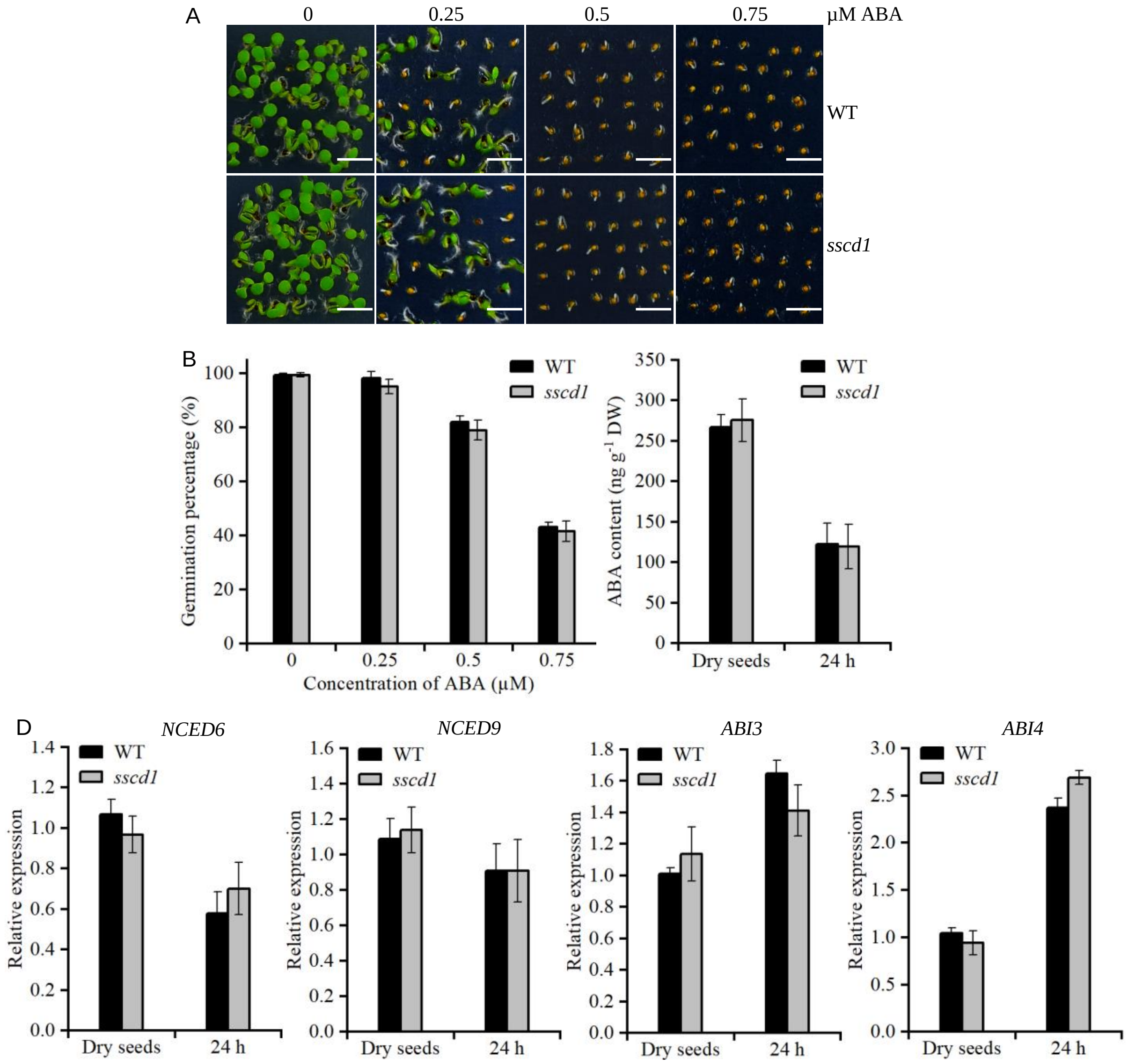

Figure S1. Seed germination of WT and *sscd1* in response to ABA. (A) Germination phenotypes of WT and *sscd1* seeds stored for 15 days on the 4th day after stratification in the presence of different ABA concentrations. Scale bars = 2.78 mm. (B) Germination percentages of WT and *sscd1* seeds described in (A). Seeds were pooled from at least 10 plants, and at least 50 seeds per genotype were used in each replicate. (C) ABA contents in dry seeds and seeds imbibed for 24 h on MS medium from WT and *sscd1* plants. (D) Relative expression of genes in the ABA pathway in WT and *sscd1* seeds is described in (C). *NCED6*, *9-cis-epoxycarotenoid dioxygenase 6*. *NCED9*, *9-cis-epoxycarotenoid dioxygenase 9*. *ABI3*, *ABA INSENSITIVE 3*. *ABI4*, *ABA INSENSITIVE 4*. Data represent the mean ± SE from three biological replicates.

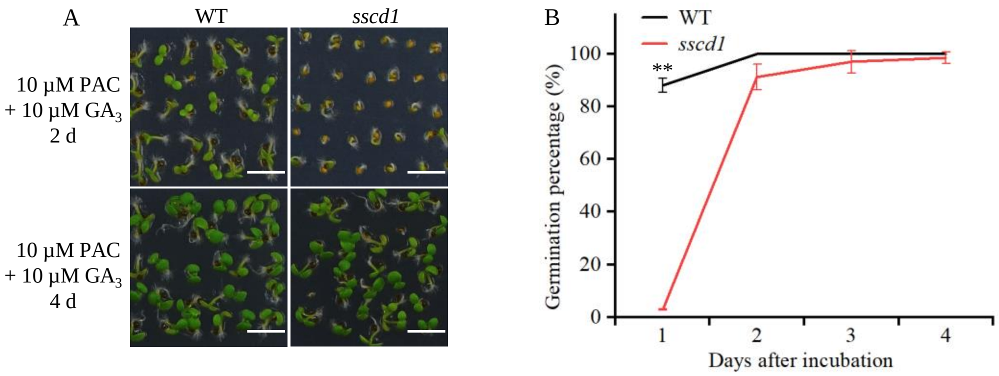

Figure S2. Germination of WT and *sscd1* seeds after 60 days of storage in the presence of 10 μM PAC and 10 μM GA_3. (A) Germination phenotypes of WT and *sscd1* seeds on the 2nd and 4th day after stratification. Scale bars = 2.78 mm. (B) Germination percentages of WT and *sscd1* seeds. Data represent the mean ± SE from three biological replicates. Asterisks represent significant differences between WT and *sscd1* (*t*-test, ***p* < 0.01). Seeds were pooled from at least 10 plants, and at least 50 seeds per genotype were used in each replicate.
