# Supplementary material for: Fumarylacetoacetate Hydrolase Regulates Seed Dormancy and Germination Through the Gibberellin Pathway in Arabidopsis
Source: Plants (Basel). 2025 Oct 31;14(21):3342. doi: 10.3390/plants14213342 (PMC12608377; doi:10.3390/plants14213342)
Supplement: Supplementary file 1 [file plants-14-03342-s001.zip › Supplementary Table S1.pdf]

Table S1 List of primers used in this study

Characterization of plant lines

Gene	Primer name	Primer sequence (5′ to 3′)
SSCD1	SSCD1 F1	CCTCGTCCTGCCGTCGCTAT
	SSCD1 R1	CTTGTGGATGGCCCTGACCT
RGL2	RGL2LP	AACCAAATCACAACAAAGACCC
	RGL2RP	CGAATCTGAATTCTCCGTCTG
	RGL2BP	TCAAACAGGATTTTCGCCTGCT

qRT-PCR analyses

Gene	Primer name	Primer sequence (5′ to 3′)
SSCD1	SSCD1 F2	GACTCGCACTTCCCTATCCAG
	SSCD1 R2	GACCATCGA AAAGCCCAGCT
NCED6	NCED6 F	GGTTAGTTACAGCTGCCGGT
	NCED6 R	GTCGAGCTAGACCGGAATGG
NCED9	NCED9 F	TGTTCGTTCACGACGAGGAG
	NCED9 R	ACGTTCCGTGGAAACCGTAA
ABI3	ABI3 F	GGATTGTGGAATACTCGGTG
	ABI3 R	TCAGGAAGCGTTGGGAGA
ABI4	ABI4 F	GTCCAGATGGGACAATTCCAACACC
	ABI4 R	CCCTAACGCCACCTCATGATGAAAC
RGL2	RGL2 F	TCGTCTTCCTCGATAGGTTCAACGA
	RGL2 R	GCCGCAACAACGTTGAGTATCTG
GA20ox1	GA20ox1 F	GATCCATCCTCCACTTTAGA
	GA20ox1 R	GTGTATTCATGAGCGTCTGA
GA20ox2	GA20ox2 F	ACCGAGACTATTTCCGAGGATT
	GA20ox2 R	TGTTTGGCATGGAGGATAATG
GA3ox1	GA3ox1 F	TCCGAAGGTTTCACCATCACT
	GA3ox1 R	TCGCAGTAGTTGAGGTGATGTTG
GA3ox2	GA3ox2 F	GTTTCACCGTTATTGGCTCTCC
	GA3ox2 R	TCACAGTATTTGAGGTGGTGGC
GA2ox2	GA2ox2 F	CCGGTTCTCACTTCCCATT
	GA2ox2 R	GCTTCCGGATCGGCTAG
GA2ox6	GA2ox6 F	GGGACAGAAGTCTAGCGAAGTG
	GA2ox6 R	TCGCTACGAACGTCTCTGATC
EXPA1	EXPA1 F	AAGAACTGGATGGCAAGCGA
	EXPA1 R	TGCTTCTACTGTGAAGGTCTGG
EXPA8	EXPA8 F	CACCATGGGCGGAGCTT
	EXPA8 R	CCACCTCGGGTCATCGTTAC
EXPA9	EXPA9 F	ACTGGCAATCCAACGCACTTCT
	EXPA9 R	TCAGACGCGGAAGTTCTTGC
ACTIN2	ACT2 F	AGCACTTGCACCAAGCAGCATG
	ACT2 R	ACGATTCCTGGACCTGCCTCATC
